# Vitamin-D Binding Protein Gene Polymorphisms and Serum 25-Hydroxyvitamin-D in a Turkish Population

**DOI:** 10.3390/metabo11100696

**Published:** 2021-10-12

**Authors:** Lutfiye Karcıoğlu Batur, Ahmet Özaydın, Murat Emrah Maviş, Gökçe Göksu Gürsu, Laurence Harbige, Nezih Hekim

**Affiliations:** 1 Department of Molecular Biology and Genetics, Faculty of Engineering and Natural Sciences, Biruni University, Istanbul 34010, Turkey; lbatur@biruni.edu.tr; 2 Department of Medical Genetics, Cerrahpasa Faculty of Medicine, Istanbul University-Cerrahpasa, Istanbul 34098, Turkey; aozaydin@istanbul.edu.tr; 3 Sem Laboratuar Cihazlari Pazarlama San. ve Tic. Inc., R&D Center, Istanbul 34746, Turkey; murat.mavis@jasem.com.tr (M.E.M.); gokce.goksu@jasem.com.tr (G.G.G.); 4 Lipidomics and Nutrition Research Centre, School of Human Sciences, London Metropolitan University, London N7 8DB, UK; l.harbige@londonmet.ac.uk

**Keywords:** 25(OH)D, Turkish population, DBP, GC gene, free vitamin D

## Abstract

The rs7041 and rs4588 polymorphisms found in the GC gene, encoding vitamin D-binding protein (DBP), have distinct biochemical phenotypes. The aim of this study was to investigate vitamin D parameters with these polymorphisms, in individuals with possible vitamin D deficiency. The most common (49% of the cohort) genotype in rs7041 was GT, especially among individuals with high levels of free 25(OH)D calculated but with low levels of bioavailable 25(OH)D, and in rs4588 it was AC in particular among the individuals with low levels of bioavailable 25(OH)D. The most common phenotypes were Gc1s/2 (35.3%) and Gc1s/1s (31.4%), and Gc1f/1f was rare (5.9%). The variations in free and bioavailable 25(OH)D levels among healthy Turkish individuals may be attributed to the variations in total 25(OH)D as well as GC gene polymorphisms. The Turkish population shares a similarity for allele frequencies of rs7041 with the European population and similarity for allele frequencies of rs4588 with Gujarati Indians, and this may also be important in relation to certain ethnic populations showing associations between vitamin D and COVID-19.

## 1. Introduction

Vitamin D can be found in the serum as bound or unbound forms [1]. Eighty-five percent to 90% of circulating 25-hydroxyvitamin D [25(OH)D] is normally bound to vitamin D-binding protein (DBP) [2], and 10% to 15% is bound to albumin (ALB). Only 1% is freely available in blood. The total of ALB-bound and free 25(OH)D forms represent bioavailable 25(OH)D [3,4,5].

Several gene variants of proteins involved in the synthesis, transportation, and activation of vitamin D have been associated with changes in serum 25(OH)D including the group-specific complement (GC) gene, which encodes DBP [6,7,8].

Combinations of rs7041 and rs4588 genotypes can identify three phenotypic groups of DBP, namely, Gc1s, Gc1f, and Gc2, thought to vary in affinity for 25(OH)D [9]. These variants are Gc1f (1f): rs7041 (T) + rs4588 (C); Gc1s (1s): rs7041 (G) + rs4588 (C); and Gc2 (2): rs7041 (T) + rs4588 (A) [9]. Since there are two copies of the GC gene, this enables the generation of six possible DBP phenotypes (Gc1f-1s, Gc1f-1f, Gc1f-2, Gc1s-2, Gc1s-1s, Gc2-2) that can be identified from the genotypes. Associations between various SNPs in this gene and the serum 25(OH)D concentrations have been investigated previously [7,8,10]; however, no study has examined this association in a Turkish population.

Low dietary vitamin D intake and/or lack of vitamin D supplements are associated with a 2-fold to 5-fold increased risk for vitamin D deficiency (<20 ng/mL). The prevalence of vitamin D deficiency varies based on how deficiency is defined (<20 vs. ≤30 ng/mL) [11]. The Institute of Medicine (IOM) concluded that 20 ng/mL was the level necessary for good bone health for practically all individuals [12].

Vitamin D deficiency is reported to be common worldwide, but little has been reported about the vitamin D status of adults in Turkey. There is a study reporting that adults living in an urban, non-coastal setting in Turkey have a high prevalence of vitamin D deficiency [13]. The aim of this study was to determine the underlying reason of a high incidence of vitamin D deficiency in Turkish healthy adult individuals who had a low serum concentration of total 25(OH)D, and to investigate whether this low level of 25(OH)D may be due to the polymorphisms in the GC gene encoding the DBP protein carried by the patient. We hypothesized that the measurement of free 25(OH)D may provide better insight into the exact prevalence of vitamin D deficiency in relation to the reported frequencies of polymorphisms in the GC gene in a Turkish cohort.

## 2. Results

Fifty-one individuals (25 male and 26 female) with a mean age of 39.39 ± 12.30 years were recruited for the study. None of the subjects had used any vitamin D supplements in the last 2 years up to the time of collecting blood samples for the study. The mean concentration of serum 25(OH)D measured by CMIA was 20.75 ± 14.2 ng/mL and by LC-MS/MS was 19.92 ± 12.5 ng/mL, which were not significantly different (*p* > 0.05). The mean concentrations of ALB and DBP of all patients were 43.82 ± 2.15 g/L and 390.24 ± 132.5 mg/L, respectively. The serum concentrations of free and bioavailable 25(OH)D were calculated as 4.28 ± 2.47 pg/mL and 1.74 ± 1.0 ng/mL, respectively.

### 2.1. Comparison of Biochemical Data and Genotype Distribution in rs7041

The three genotypes (GG, GT, and TT) of the rs7041 polymorphism were in Hardy–Weinberg equilibrium. The most common genotype was found to be GT (49%) and the least common was TT (20%). The frequency of the GG genotype was 31%.

The mean levels of serum 25(OH)D and free and bioavailable 25(OH)D were significantly lower in individuals with GT (by 37–39%) and TT genotypes (by 46–53%) compared with the GG genotype (*p* < 0.01). However, there was no significant difference between the three groups in terms of DBP concentration (*p* > 0.05), (Table 1).

When the genotype distribution and allele frequencies were compared according to the low (<2 pg/mL) and high (>2 pg/mL) levels of free 25(OH)D, there was no difference between the three genotypes (*p* > 0.05), except the number of individuals with the GT genotype who had high levels of free 25(OH)D, which was significantly higher than the group with low levels (*p* < 0.05). The T allele was the most common allele in the group with low free 25(OH)D levels, whereas the G allele was common in the group with a low level, but this was not significant (*p* > 0.05), (Table 2).

Comparing the groups comprised of low (<2 ng/mL) and high (>2 ng/mL) levels of bioavailable 25(OH)D (Table 2), the difference in the number of individuals with GG and GT genotypes was significant (*p* < 0.05), whereas there was no statistically significant difference found for individuals with the TT genotype (*p* > 0.05). The allele frequencies of G were found to be higher among individuals with a high level of bioavailable D vitamin while the low group (<2 ng/mL) had similar allele frequencies between G and T genotypes, which were not significant (*p* > 0.05) (Table 2).

**Figure 1:**
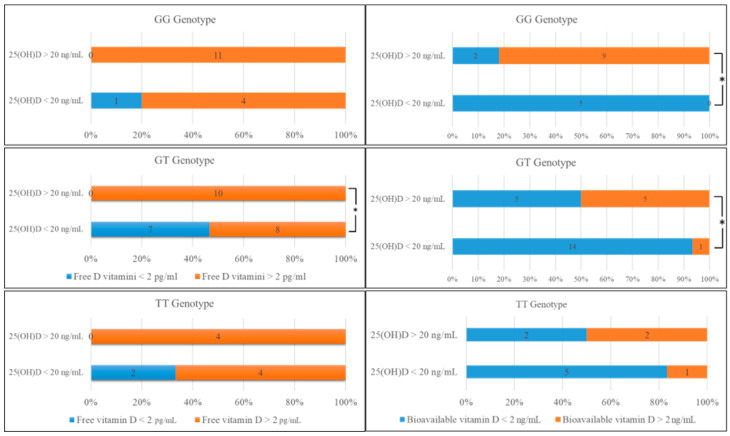
Comparison of the number of individuals with GG, GT, and TT genotypes according to serum 25(OH)D levels compared with the free and bioavailable 25(OH)D levels. * *p* < 0.05 indicates the significance level.

For all genotypes, all individuals with normal concentrations of 25(OH)D had high levels of free 25(OH)D, whereas some individuals with low concentration of 25(OH)D had a high level of free or bioavailable 25(OH)D (Figure 1).

### 2.2. Comparison of the Biochemical Data and Genotype Distribution in rs4588

Among three genotypes (AA, AC, and CC) of rs4588 polymorphism, the most common genotype was found to be AC (53%), the second was CC genotype (45%), and the least common was AA (2%) among individuals (Table 3).

There was only one individual in the AA genotype group, who was, therefore, excluded from statistical analysis. Individuals with AC genotype had a lower mean of 25(OH)D and free and bioavailable 25(OH)D values compared with the levels of the CC group (*p* < 0.05) (Table 3).

When the distribution of genotypes and allele frequencies were compared according to the level of free 25(OH)D, a significantly higher percentage of individuals with AC and CC genotypes had normal levels of free 25(OH)D (*p* < 0.05). There was considerable difference between the groups with low and high levels of bioavailable 25(OH)D among the AC and CC genotypes (*p* < 0.05) (Table 4).

**Figure 2:**
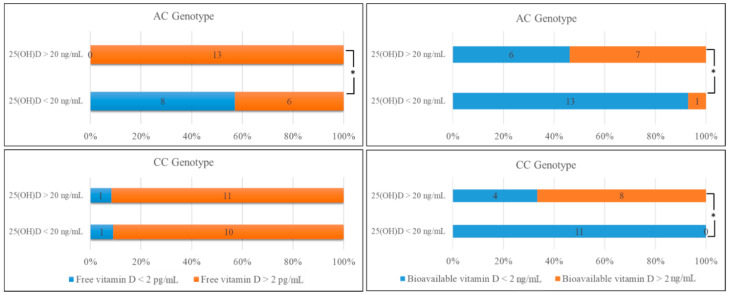
Comparison of the number of individuals with AC and CC genotypes according to serum 25(OH)D levels compared with the free and bioavailable 25(OH)D levels. * *p* < 0.05 indicates the significance level.

Comparison of the mean levels of 25(OH)D and free 25(OH)D in the AC genotype showed that free 25(OH)D levels were high in all subjects with normal serum 25(OH)D concentrations (Figure 2). Most of the individuals with low 25(OH)D concentrations also had low levels of free 25(OH)D, which was significantly different (*p* < 0.05). On the other hand, nearly half of the individuals with normal serum 25(OH)D concentration were found to have low levels of bioavailable 25(OH)D with the rest of the subjects having high values. In addition, most of the individuals with low 25(OH)D concentration showed a low level of bioavailable 25(OH)D (*p* < 0.05), (Figure 2).

Comparing the free 25(OH)D levels in the CC genotype, most (92%) of the individuals with normal 25(OH)D concentrations showed a high level of free 25(OH)D; moreover, most (91%) of the individuals with low 25(OH)D concentrations also showed high values of free 25(OH)D, although not statistically significant (Figure 2). Bioavailable 25(OH)D levels for the CC genotype indicated that two-thirds of the individuals with normal 25(OH)D concentrations had higher levels of bioavailable 25(OH)D. For all subjects with low 25(OH)D concentration, the level of bioavailable 25(OH)D was also significantly low (*p* < 0.05), (Figure 2).

### 2.3. Comparison of Biochemical Data According to DBP Phenotypes

Determining the phenotypes of individuals with a combination of rs7041 and rs4588 polymorphisms, the most common phenotypes were Gc1s/2 (35.3%) and Gc1s/1s (31.4%), and the rare phenotypes were Gc1s/1f (13.7%), GC1f/2 (13.7%), and Gc1f/1f (5.9%) (Table 5).

The mean concentration of 25(OH)D and free and bioavailable 25(OH)D levels of individuals with Gc1s/2 and Gc1f/2 phenotypes were significantly lower than the other phenotypes (*p* < 0.05 and *p* < 0.01, respectively). Importantly, the concentration of DBP did not show any significant difference among the five phenotypes (*p* > 0.05) (Table 5).

The distribution of phenotypes compared according to the concentrations of 25(OH)D and free and bioavailable 25(OH)D levels showed Gc1s/2 to be the most common phenotype among individuals with low concentrations of 25(OH)D and free 25(OH)D, whereas GC1s/1s was the most common phenotype among individuals with normal concentrations of the vitamins (*p* < 0.05). Additionally, Gc1f/1f was the least common phenotype for both groups with normal and low levels of 25(OH)D, was the least common phenotype with higher levels of free 25(OH)D, and was the group with relatively low levels of bioavailable 25(OH)D (Table 5). Gc1s/1f and Gc1f/2 were the rarest phenotypes among individuals with high levels of bioavailable 25(OH)D (Table 6).

## 3. Discussion

The GC gene is highly polymorphic. Gc1f, Gc1s, and Gc2 correspond to different allele arrangements of the two SNPs commonly found in the GC gene (rs7041 and rs4588), and these have been associated with a variety of disorders and deficiencies including vitamin D deficiency [14,15]. Since 90% of circulating 25(OH)D is closely bound to DBP, the different isoforms may affect the serum concentration and the bioavailability of 25(OH)D [16]. However, it is not known how and to what extent these isoforms affect the region where serum 25(OH)D binds. Some polymorphisms have been shown to lead to faster release of bound vitamin D, while others have been shown to be slower at releasing vitamin D [17]. According to the free hormone hypothesis, only free 25(OH)D (not the bound one) can enter cells to be converted to 1,25(OH)2D, which is the active form of vitamin D. The total 25(OH)D concentration includes vitamin D bound to DBP or ALB plus the free 25(OH)D, all of which determine the total biological vitamin D. The role of DBP, which serves as a bound reservoir for serum 25(OH)D, appears to be to extend the half-life of 25(OH)D and limit the availability of free 25(OH)D [18].

To our knowledge, this is the first study that describes the interaction between vitamin D-related polymorphisms and free and bioavailable 25(OH)D levels, giving new biochemical insight about these physiological parameters in a Turkish population. However, this interaction needs more advance studies comparing different ethnicities in different populations to further elucidate the association between vitamin D-related polymorphisms and free and bioavailable 25(OH)D levels and in relation to vitamin D deficiency.

In the present study, rs4588 and rs7041 polymorphisms were selected due to their high prevalence among the Turkish population and their significant associations with DBP functions. The comparison of vitamin D parameters for the rs4588 polymorphism revealed that the individuals with heterozygous polymorphism (AC) had significantly lower levels of 25(OH)D, free 25(OH)D, and bioavailable 25(OH)D compared with homozygous polymorphic (CC) individuals, and these were independent of DBP concentration, which did not vary significantly between the genotype groups. Our results suggest that clinically measured 25(OH)D levels in individuals with the C allele do not always comply with free and bioavailable 25(OH)D levels, and there seems to a relation between the allele frequency of rs4588 with the incidence of low levels of bioavailable 25(OH)D in the Turkish population. The fact that the number of individuals having low total 25(OH)D concentration in all genotypes was lower than the patients with low free 25(OH)D levels explains why vitamin D deficiency has been diagnosed more frequently due to measurement of total 25(OH)D levels only (and not free 25(OH)D) clinically in the Turkish population [13].

In DBP gene polymorphisms in other populations, rs4588 and rs7041 polymorphisms were most consistently associated with lower 25 (OH)D levels [19,20,21,22,23]. There are also other variants of GC, namely, rs16846876, rs17467825, rs2282679, rs3755967, rs2298850, and rs1155563, which were reported to be significantly associated with both 25(OH)D concentrations and vitamin D deficiency (25(OH)D < 15 ng/mL) [24]. However, there are also controversial results of some studies that claimed that both SNPs were not associated with lower levels of 25 (OH)D [25]. These differences may be explained by ethnic differences that are related to variations in 25 (OH)D concentrations, parathyroid hormone concentration, and calcium homeostasis [26].

Many studies have found vitamin D deficiency (as defined by a 25(OH)D level of less than <20 ng/mL) in 40–100% of those tested, with proportions varying according to geographic area, latitude, and specifics of the patient population [27,28,29,30]. In a study by Powe et al., the mean serum concentrations of 25(OH)D were lower in Black Americans compared to White Americans, suggesting vitamin D functions, such as maintaining bone density and decreasing bone desorption and fractures, would be expected to be compromised in Black Americans, but surprisingly it is the reverse [1]. The current study and other long-term studies measuring 25(OH)D levels have highlighted the role of DBP since it was revealed that the mean concentration of DBP in Blacks is lower (168 ± 3 µg/mL) than the concentration in whites (337 ± 5 μg/mL) [31]. Interestingly and surprisingly, in a population-based study in Turkey, a high prevalence of vitamin D deficiency (74.9%) and insufficiency (13.8%) was found in an area of the country that gets lots of sunlight [13]. The data from this present study suggest that the underlying reason for diagnosing a high prevalence of vitamin D deficiency in the above study in a Turkish population may be linked to the allele frequencies of rs7041 and rs4588. However, other reasons, including lack of adequate sunlight exposure or excessive use of sunscreen and/or inadequate dietary intake of vitamin D, may also have contributed to the findings.

There are controversial data claiming that the differences in total 25(OH)D among populations or ethnicities depend on the derivation of free 25(OH)D using results obtained from a monoclonal assay for calculating DBP concentration [23]. Nielson et al. showed that the monoclonal ELISA underestimates DBP concentrations in individuals with GC/1f alleles [32]. However, when DBP was assessed using immunoassays of DBP with polyclonal antibodies and proteomic methods, these GC-dependent differences were not apparent and in line with other proteomic data [33]. In the present study, we used a widely used sensitive monoclonal immunoassay for DBP measurement [34] and found that the concentration of DBP did not show discrepancy among the five phenotypes of DBP. To fully confirm our preliminary observations on the association between DBP concentrations and genotypes in a Turkish population, it is necessary to investigate a much larger number of healthy Turkish individuals.

We aim to measure the free 25(OH)D by ELISA and use this method to establish the clinical relevance of free 25(OH)D with genetic variations [35,36].

A study conducted in Norway showed that the serum 25(OH)D levels in individuals with Gc1/1s, Gc1s/1f, and Gc1f/1f phenotypes were found to be in the normal range (>50 nmol/L) [37]. They also found that Gc1s/2 and Gc1f/2 phenotypes had similar limits of 50 nmol/L, but Gc2/2 showed a lower level of serum 25(OH)D. In the present study, the average of 25(OH)D levels in the phenotypes Gc1s/1s, Gc1s/1f, and Gc1f/1f were 69.1 ± 34.2 nmol/L, 43.11 ± 10.7 nmol/L, and 60.55 ± 32.5 nmol/L (all values converted to nmol/L), respectively, and no significant difference was found. However, Gc1s/1f and Gc1f/2 phenotypes showed significantly decreased values, i.e., 41.1 ± 23.5 nmol/L and 32.7 ± 16.2 nmol/L, respectively. Thus, Gc1s/1f, Gc1s/1f, and Gc1f/2 phenotypes appear to be more prone to vitamin D deficiency in this Turkish population. This may be dependent on the heterogeneity resulting from the rs4588 polymorphism, rather than the rs7041 polymorphisms, and, therefore, related to the low levels of free and bioavailable 25(OH)D.

The relationships between rs7041 and rs4588 polymorphisms and serum 25(OH)D concentrations have been previously investigated in Scandinavian children, where significant differences were observed in serum 25(OH)D concentrations between the phenotypes [38]. Although the main predictor of free 25(OH)D is total 25(OH)D and vitamin D deficiency is clinically diagnosed by total 25(OH)D, the findings of the present study support the suggestion that if the level of free 25(OH)D is examined in a Turkish population, the diagnosis of vitamin D deficiency would be expected to be reduced. In addition, if the cutoff values for the free 25(OH)D could be measured or GC gene polymorphisms could be routinely determined (despite being an expensive analysis), vitamin D supplementation may be unnecessary in some patients who are deficient in 25(OH)D measured in serum, due to the variations in vitamin D binding to DBP protein resulting from GC gene polymorphisms.

Another important finding of our study was that there were some similarities between our Turkish population with others in terms of DBP polymorphisms. In contrast to the AA genotype being the rarest genotype among Turks, according to a study by Powe et al., the AA genotype was the most common genotype in a Black population having rs4588 polymorphism [1]. The AA genotype was seen in only one individual in the current study; thus, it can be concluded that the heterogeneity of rs4588 SNP is high in the Turkish population. Moreover, the allele frequencies of rs4588 in the Turkish population were similar to the allele frequencies for Gujarati Indians, the only population with a higher frequency of AC genotype than the CC genotype. Furthermore, Petersen et al., showed that the distributions of GG, GT, and TT alleles are 33%, 46%, and 21% and CC, AC, and AA alleles are 51%, 41%, and 51% in the European population, respectively [10], suggesting that the Turkish population is similar to the European population in terms of rs7041 polymorphism.

Finally, our current observations may be highly significant in relation to the SARS-CoV-2 pandemic/COVID-19 disease and the role of vitamin D. We recently reported the highest prevalence of COVID-19 among Black people previously found 25(OH)D deficient, compared with Whites, Chinese Asians, and Asian Indians in the UK [39].

## 4. Materials and Methods

### 4.1. Patient Selection

This prospective study was conducted with 51 healthy adult individuals older than 18 years old and from both genders, who applied to one institute for a routine annual check-up, met the study criteria, and agreed to participate in the study. The selection criteria were not using any vitamin D supplements for the last 2 years, not having any health problem such as obesity that would affect the vitamin D concentrations, and being of Turkish ethnic origin. To determine the sample size and to conduct power analysis for examination of association between the vitamin D parameters and the polymorphisms of healthy individuals, GPower analysis was used. The effect size for distribution of polymorphisms was determined as 0.3 from a pilot application. With 0.01 Type I error rate, 0.3 effect size, and 81% power, the minimum needed number of cases was determined as 51 for total analysis.

The study was approved by Biruni University Non-Interventional Research Ethics Committee (Approval No: 2017/10-1). The planning, conduct, and reporting of this research were in accordance with the Helsinki Declaration as revised in 2013. The written consent forms were obtained from all subjects who were informed about the study.

### 4.2. Blood Samples

Venous blood samples were collected into one EDTA tube and one serum tube containing a gel (Nest- UK). Blood in serum tubes was centrifuged at 4100 rpm (NF 800, Nuve, Turkey), divided into three aliquots, and frozen at −80 °C. Whole blood samples in EDTA were stored at +4 °C until the DNA isolation.

### 4.3. Measurement of Serum 25(OH)D, Albumin, and Vitamin D-Binding Protein Concentrations

Total 25(OH)D concentrations were measured by a chemiluminescence microparticle immunoassay method (CMIA), using an Architect 25-OH Vitamin D kit (5P02, Abbott Diagnosis, Chicago, IL, USA) and an i1000SR analyzer (Abbott Laboratories, Chicago, IL, USA). The cutoff value was determined as 20 ng/mL, according to a 2011 report on dietary reference intakes for vitamin D from the IOM [12].

ALB concentrations were measured by a colorimetric test study (Roche/Hitachi Cobas C, Mannheim, Germany), according to the procedure of the manufacturer.

DBP concentration in serum was measured according to the procedure using a quantikine human vitamin DBP monoclonal immunoassay kit (Catalogue number DVDBP0, R&D Systems, Minneapolis, MN, USA), according to the literature [40].

### 4.4. LC-MS/MS Method

Analysis of 25(OH)D3 and vitamin D2 were performed using an Agilent Infinity 1290 HPLC system (Agilent Technologies, Santa Clara, CA, USA), consisting of a binary pump system (G4220A), column compartment (G1316C), and autosampler (G7167B) coupled with 6470 triple quadrupole mass spectrometer (6470A, Agilent Technologies, Santa Clara, CA, USA) using CE-in vitro diagnostic certified Jasem vitamin D LC-MS/MS analysis kit (Sem Laboratuar Cihazları Pazarlama, Istanbul, Turkey). Serum-based calibrants/quality control material and patient samples were prepared in accordance with the kit sample preparation protocol including a protein precipitation step prior to injection. The HPLC system was operated according to the kit chromatographic method settings, and positive electronic spray ionization in multiple-reaction monitoring mode was implemented for the MS/MS detection. The peak area ratio of the 25(OH)D3 and vitamin D2 to the assigned internal standard (labeled stable isotope-d6 25(OH)D3) was assessed for the accurate determination of analyte concentration.

### 4.5. DNA Isolation

DNA isolation from whole blood was performed using a quick-DNATM miniprep plus (Zymo Research, Irvine, CA, USA) DNA isolation kit. Isolated DNAs were stored at −200 °C until further analysis. DNA quality and concentration measurements were performed using 2 µL DNA with a NanoDrop 2000c spectrophotometer (Thermo fisher Scientific, Gaithersburg, MD, USA).

### 4.6. Real-Time PCR

Using isolated, genomic DNA, genotyping for the most common SNPs of DBP, rs4588 and rs7041, was performed by using TaqMan probes for real-time PCR. The SNP assay coded by C_8278879_10 (Applied Biosystems TaqMan SNP Genotyping Assays Thermo Fisher Scientific, Gaithersburg, MD, USA) was used for genotyping rs4588, and SNP assay coded by C_3133594_30 (Thermo Fisher Scientific, Gaithersburg, MD, USA) was used for genotyping rs7041. The base sequences were

CTTGTTAACCAGCTTTGCCAGTTCC*[G/T]TGGGTGTGGCATCAGGCAATTTTC and CTTTGCCAGTTCCGTGGGTGTGGC*[A/C]TCAGGCAATTTTGCTTTTAGTCGT, respectively. Real-time temperature cycle reaction conditions were adjusted according to the protocol of the manufacturer and Peršić et al. [41].

### 4.7. Calculation of Free 25(OH)D Levels

Free 25(OH)D was calculated by using the following Equation [1], which is also known as the Bikle Method:

Free\25(OH)D\ = Total 25(OH)D1 + (Kalb [Alb]) + (KDBP [VDBP])

Bioavailable D vitamin was calculated by summing the concentration of free 25(OH)D with ALB-bound vitamin D [18]. The reference range of free and bioavailable 25(OH)D levels were determined as 2 pg/mL and 2 ng/mL, respectively, according to the report by Aloia et al. [18].

### 4.8. Statistical Analysis

Statistical analysis of the data was performed by using GraphPad InStat ver. 3.06 (GraphPad Inc, San Diego, CA, USA). The Kolmogorov–Smirnov distance test was used to test the normality of all in-group variables.

A paired t-test or unpaired t-test with Welch correction was used for comparison of the numerical variables with normal distribution, and the Mann–Whitney test or Wilcoxon matched pairs signed-ranks test were used for not normally distributed variables. For multiple comparison of three or more groups, one-way analysis of variance (ANOVA) and post hoc Tukey–Kramer Multiple Comparisons Test were performed for normally distributed variables, and the Kruskal–Wallis Test (Nonparametric ANOVA) and Dunn Multiple Comparison test were used for other variables. A Chi-squared test was also used to compare the categorical variables of two groups and to assess whether the SNPs were in Hardy–Weinberg equilibrium in the sample tested.

## 5. Conclusions

In the Turkish population cohort studied, the most common (49% of the cohort) genotype in rs7041 was GT, particularly among individuals with high levels of calculated free 25(OH)D, but with low levels of bioavailable 25(OH)D. In rs4588, it was AC, particularly among individuals with low levels of bioavailable 25(OH)D. The most common phenotypes were Gc1s/2 (35.3%) and Gc1s/1s (31.4%), and Gc1f/1f was rare (5.9%). The variations in free and bioavailable 25(OH)D levels among healthy Turkish individuals may be attributed to the variations in total 25(OH)D as well as GC gene polymorphisms. It is, therefore, suggested that the levels of free and bioavailable 25(OH)D (and gene polymorphisms) should be examined clinically among healthy Turkish individuals, in addition to the conventional measurement of serum 25(OH)D.

Finally, our current observations may be highly significant in relation to the SARS-CoV-2 pandemic/COVID-19 disease and the role of vitamin D. Recently one of our co-authors reported the highest prevalence of COVID-19 among Black people, previously found 25(OH)D deficient, compared with Whites, Chinese Asians and Asian Indians in the UK.

## Figures and Tables

**Table 1:** Comparison of vitamin D parameters within the three genotype cohorts of individuals with the rs7041 polymorphism.

	Genotypes X ± SD			*p* Values
	GG (*n* = 16)	GT (*n* = 25)	TT (*n* = 10)	
LC-MS/MS-25(OH)D (ng/mL)	27.68 ± 13.69	17.33 ± 10.71 *	12.95 ± 5.52 *	0.005
DBP (mg/L)	391.83 ± 91.10	409.64 ± 162.41	337.4 ± 98.45	0.134
Free 25(OH)D (pg/mL)	5.73 ± 2.58	3.62 ± 2.20 *	2.74 ± 1.36 *	0.006
Bioavailable 25(OH)D (ng/mL)	2.35 ± 1.03	1.43 ± 0.85 *	1.27 ± 0.65 *	0.004

* *p* < 0.01 vs. GG genotype.

**Table 2:** Genotype distributions and allele frequencies of individuals with rs7041 polymorphism, grouped according to free and bioavailable 25(OH)D levels.

*n* (%)		Free 25(OH)D < 2 pg/mL (*n* = 10)	Free 25(OH)D > 2 pg/mL (*n* = 41)	*p* Values	Bioavailable 25(OH)D < 2 ng/mL (*n* = 33)	Bioavailable 25(OH)D > 2 ng/mL (*n* = 18)	*p* Values
Genotype distribution	GG	1 (6)	15 (94)	0.052	7 (43.75)	9 (56.25) *	0.017
	GT	7 (28)	18 (72) *	0.033	19 (76)	6 (24) *	0.049
	TT	2 (20)	8 (80)	0.486	7 (70)	3 (30)	0.348
Allele frequency	G	0.80	0.80	0.259	0.79	0.83	0.155
	T	0.90	0.63		0.79	0.50	

* *p* < 0.05 vs. the patients with free 25(OH)D level < 2 pg/mL or with bioavailable 25(OH)D < 2 ng/mL.

**Table 3:** Comparison of vitamin D parameters within two genotype cohorts of individuals with the rs4588 polymorphism.

	Genotypes X ± SD		
	AC (*n* = 27)	CC (*n* = 23)	*p* Values
LC-MS/MS-25(OH)D (ng/mL)	16.03 ± 8.91	24.58 ± 13.04 *	0.032
DBP (mg/L)	372.28 ± 144.83	409.82 ± 119.30	0.150
Free 25(OH)D (pg/mL)	2.99 ± 1.76	4.35 ± 2.53 *	0.043
Bioavailable 25(OH)D (ng/mL)	1.41 ± 0.81	2.09 ± 0.90 *	0.017

* *p* < 0.05 vs. AC genotype. Only one individual carried the CC genotype and was excluded from the analysis.

**Table 4:** Genotype distributions and allele frequencies of individuals with rs4588 polymorphism, grouped according to free and bioavailable 25(OH)D levels.

*n* (%)		Free 25(OH)D < 2 pg/mL (*n* = 10)	Free 25(OH)D > 2 pg/mL (*n* = 41)	*p* Values	Bioavailable 25(OH)D < 2 ng/mL (*n* = 33)	Bioavailable 25(OH)D > 2 ng/mL (*n* = 18)	*p* Values
Genotype distribution	AA	0 (0)	1 (%100)	0.309	0 (0)	1 (100)	N/A
	AC	8 (%30)	19 (%70) *	0.028	19 (70.4)	8 (29.6)	0.276
	CC	2 (%9)	21 (%91) *	0.038	15 (65.2)	8 (34.8)	0.421
Allele frequency	A	0.80	0.49	0.194	0.56	0.53	0.495
	C	1.00	0.98		1.0	0.94	

* *p* < 0.05 the patients with free 25(OH)D level < 2 pg/mL. N/A: Not applicable.

**Table 5:** Comparison of the phenotypes of GC gene according to biochemical parameters.

Phenotypes	X ± SD			
	Total 25(OH)D (ng/mL)	DBP (mg/L)	Free 25(OH)D (pg/mL)	Bioavailable 25(OH)D (ng/mL)
Gc1s/1s (*n* = 16)	27.69 ± 13.7	391.8 ± 91.1	5.42 ± 2.35	2.44 ± 0.99
Gc1s/1f (*n* = 7)	17.27 ± 4.3	423.4 ± 163.8	3.28 ± 0.93	1.55 ± 0.65
Gc1s/2 (*n* = 18)	16.48 ± 9.4 *	361.7 ± 88.6	2.56 ± 1.46 **	1.34 ± 0.79 **
Gc1f/1f (*n* = 3)	24.26 ± 13.0	417.0 ± 153.8	4.92 ± 2.62	2.05 ± 1.17
Gc1f/2 (*n* = 7)	13.10 ± 6.5 *	303.3 ± 46.1	2.48 ± 1.26 **	1.19 ± 0.59 **
*p* value	0.016	0.202	0.003	0.005

* *p* < 0.05, **p < 0.01 vs. the patients with GC1s/1s phenotype.

**Table 6:** Distribution of the phenotypes of GC gene according to the levels of 25(OH)D and free and bioavailable 25(OH)D.

Phenotype	Total 25(OH)D < 20 ng/mL (*n* = 26)	Total 25(OH)D ≥ 20 ng/mL (*n* = 25)	*p* Values	Free 25(OH)D < 2 pg/mL (*n* = 9)	Free 25(OH)D ≥ 2 pg/mL (*n* = 42)	*p* Values	Bioavailable 25(OH)D < 2 ng/mL (*n* = 33)	Bioavailable 25(OH)D ≥ 2 ng/mL (*n* = 18)	*p* Values
Gc1s/1s	5 (31.25)	11 (68.75) *	0.028	1 (6.25)	15 (93.75)	0.074	7 (43.75)	9 (56.25)*	0.017
Gc1s/1f	5 (71.43)	2 (28.57)	0.122	0 (0)	7 (100)	0.204	6 (85.72)	1 (14.28)	0.105
Gc1s/2	10 (55.56)	8 (44.44)	0.315	6 (33.34)	12 (66.66) *	0.015	13 (61.54)	5 (38.46)	0.203
Gc1f/1f	2 (66.67)	1 (33.33)	0.288	0 (0)	3 (100)	0.204	1 (33.34)	2 (66.66)	0.121
Gc1f/2	4 (57.15)	3 (42.85)	0.363	2 (28.58)	5 (71.42)	0.207	6 (83.34)	1 (16.66)	0.105

* *p* < 0.05 vs. low levels of 25(OH)D.

## Data Availability

The data presented in this study are available within the article.
